# Complete Genome Sequences of Five Representative *Staphylococcus aureus* ST398 Strains from Five Major Sequence Heterogeneity Groups of a Diverse Isolate Collection

**DOI:** 10.1128/genomeA.00473-17

**Published:** 2017-06-08

**Authors:** Jo-Ann McClure, Kunyan Zhang

**Affiliations:** a Centre for Antimicrobial Resistance, Alberta Health Services/Calgary Laboratory Services/University of Calgary, Calgary, Alberta, Canada; b Department of Pathology & Laboratory Medicine, University of Calgary, Calgary, Alberta, Canada; c Department of Microbiology, Immunology and Infectious Diseases, University of Calgary, Calgary, Alberta, Canada; d Department of Medicine, University of Calgary, Calgary, Alberta, Canada; e The Calvin, Phoebe and Joan Snyder Institute for Chronic Diseases, University of Calgary, Calgary, Alberta, Canada

## Abstract

*Staphylococcus aureus* sequence type 398 (ST398) is a rapidly emerging livestock-associated strain causing zoonotic disease in humans. The course of pathogen evolution remains unclear, prompting whole-genome comparative studies in attempts to elucidate this issue. We present the full, annotated genomes of five newly isolated representative ST398 strains from five major sequence heterogeneity groups of our diverse isolate collection.

## GENOME ANNOUNCEMENT

*Staphylococcus aureus* multilocus sequence type (MLST) ST398 was originally reported as a livestock-associated methicillin-resistant *S. aureus* (LA-MRSA) (1, 2). Since then, this strain has become a rapidly emerging cause of human infections globally, affecting people in contact with farm animals as well as those not in contact (3–6). The ancestry and evolution of this strain are of great interest, and genome sequence-based analysis will provide insight into the relationship between animal and human lineages (7, 8). We applied whole-genome sequencing to analyze our diverse collection of ST398 isolates/strains and present here five representative complete genome sequences from five major sequence heterogeneity groups.

The genomes were sequenced with Pacific Biosciences (PacBio) RSII sequencing technology, using one single-molecule real-time (SMRT) cell. Contig assembly was performed using the Hierarchical Genome Assembly Process (HGAP) workflow (9–11) and gene annotation was performed using NCBI’s Prokaryotic Genomes Annotation Pipeline. Strain 293G generated 96,022 raw reads covering 918,615,307 sequenced bases, with an average read length of 9,566 bp (longest 52,929 bp). The estimated genome coverage was 294× and the G+C content of the resulting genome was 32.95%. One contig was generated, of 2,745,528 bp. A total of 2,832 genes were identified, of which there were 2,651 coding sequences (CDS), 82 RNA genes, and 99 pseudogenes. Strain GD5 had 101,767 raw reads covering 1,214,488,072 sequenced bases, with an average read length of 11,934 bp (longest 50,281 bp). The estimated genome coverage was 392× and the G+C content of the resulting genome was 32.93%. Two contigs resulted, one of 2,784,749 bp and one of 7,358 bp (sequence corresponds to plasmid DNA [data not shown]). A total of 2,858 genes were identified, of which there were 2,672 coding sequences (CDSs), 83 RNA genes, and 103 pseudogenes. Strain GD705 had 103,385 raw reads covering 1,232,588,292 sequenced bases, with an average read length of 11,922 bp (longest 58,954 bp). The estimated genome coverage was 400× and the G+C content of the resulting genome was 32.92%. One contig was generated, of 2,832,144 bp. A total of 2,948 genes were identified, of which there were 2,747 CDSs, 82 RNA genes, and 119 pseudogenes. Strain GD1539 had 111,049 raw reads covering 1,193,817,445 sequenced bases, with an average read length of 10,750 bp (longest 49,650 bp). The estimated genome coverage was 387× and the G+C content of the resulting genome was 33.00%. One contig was generated, of 2,819,047 bp. A total of 2,958 genes were identified, of which there were 2,765 CDSs, 82 RNA genes, and 111 pseudogenes. Strain GD1677 generated 113,141 raw reads covering 1,214,305,810 sequenced bases, with an average read length of 10,971 bp (longest 50,451 bp). The estimated genome coverage was 401× and the G+C content of the resulting genome was 32.92%. One contig was generated, of 2,813,235 bp. A total of 2,888 genes were identified, of which there were 2,694 CDSs, 82 RNA genes, and 112 pseudogenes.

### Accession number(s).

The complete genome sequences have been deposited in GenBank with accession numbers as follows: 293G, CP019591; GD5, CP019592; GD705, CP019593; GD1539, CP019594; and GD1677, CP019595.
